# A Case of Major Depressive Disorder With Peripartum Onset With Heralding Symptoms

**DOI:** 10.7759/cureus.8393

**Published:** 2020-06-01

**Authors:** Matt Narlesky, Amanda Lemp, Suporn Braaten, Robert G Wooten, Andrew Powell

**Affiliations:** 1 Psychiatry, Unity Health, Searcy, USA; 2 Psychiatry, Arkansas College of Osteopathic Medicine, Fort Smith, USA; 3 Research, Unity Health, Searcy, USA

**Keywords:** major depressive disorder with peripartum onset, peripartum depression, postpartum depression, depression after birth, postpartum mental health, mdd with peripartum onset, post-partum mood disorder

## Abstract

This report describes a case of major depressive disorder with peripartum onset in a 24-year-old African-American female who was admitted to the inpatient psychiatric facility after a suicide attempt. The patient demonstrated what we will describe as heralding symptoms during the birth of her second child, but her symptoms dramatically worsened one month prior to her admission with the birth of her third child. The patient presented with anxiety and depressive symptoms, including decreased energy, disrupted sleep patterns, and emotional lability. At the time of her admission, the patient had failed treatment with bupropion, which had been prescribed by her obstetrician. During her stay, the patient was treated with fluoxetine and participated in group therapy sessions. The patient gradually exhibited improvement in her depressive symptoms during the course of her six-day stay. At the time of discharge, the patient's depression was adequately managed with her medication regimen, and she was eager to be reunited with her newborn child. This case highlights the idiosyncratic nature of major depressive disorder with peripartum onset and the need for providers to tailor the treatment to the patient’s need.

## Introduction

According to DSM-5 (Diagnostic and Statistical Manual of Mental Disorders, 5th Edition), major depressive disorder (MDD) with peripartum onset, formerly known as postpartum depression, is characterized by MDD symptoms that begin during pregnancy or within four weeks of delivery [1]. The symptoms of MDD with peripartum onset include a depressed mood and/or anhedonia for at least two weeks plus five or more of the following: weight loss or decreased appetite, insomnia or hypersomnia, agitation or retardation, fatigue or loss of energy, feelings of excessive guilt, and a decreased ability to concentrate or thoughts of death or suicide [1]. A national survey using face-to-face interviewing in the United States estimated that 9% of postpartum women experience the disorder [2]. Clinical studies offer a less conservative estimate, reporting rates between 10% and 16% [2].

The Edinburgh Postnatal Depression Scale, a commonly used screening tool for MDD with peripartum onset, classifies the disorder as mild, moderate, or severe depending on the quantity, severity, and impact of the patient’s symptoms [2]. For example, the Edinburgh Postnatal Depression Scale defines mild-to-moderate episodes as including five to six symptoms and having a score typically between 12 and 20. Mild-to-moderate episodes normally do not present with suicidal behavior or obvious impairment in daily functioning and are generally responsive to outpatient treatment [3]. Patients experiencing a severe episode of MDD with peripartum onset present with seven to nine symptoms, including suicidal ideation and impairment in the ability to function [4]. Patients with severe MDD with peripartum onset are more likely to develop psychosis symptoms and require hospitalization [4].

Psychotherapy, such as cognitive behavioral therapy (CBT) or interpersonal psychotherapy, is the first-line therapy for women experiencing a mild-to-moderate episode of MDD with peripartum onset [3]. CBT seeks to modify the way patients think about and react to their thoughts, symptoms, and environment, whereas interpersonal therapy focuses on repairing relationships and circumstances contributing to the depressive symptoms, such as the patient’s emotions about motherhood [3].

Antidepressants are also a mainstay of treatment. Because of the danger associated with untreated MDD with peripartum onset, the benefits of treatment with antidepressants during pregnancy and/or breastfeeding generally outweigh the risks [4]. The primary approach to treating severe MDD with peripartum onset is pharmacotherapy in conjunction with psychotherapy [5]. Selective serotonin reuptake inhibitors (SSRIs) are the preferred antidepressant class in treating MDD with peripartum onset; however, if the patient was successfully treated with another antidepressant in the past, it should be continued [6]. Breastfeeding patients should be treated with low-dose monotherapy to minimize the infant’s exposure to medications [6]. Sertraline and paroxetine are the first-line agents in breastfeeding patients [6]. Although the data on the safety of SSRIs in breastfeeding women are not robust, studies of breastfeeding patients being treated with paroxetine and sertraline did not show detectable levels in the serum of breastfed infants despite detectable levels in the breast milk [6].

## Case presentation

A 24-year-old African American female presented to the inpatient psychiatric unit with self-inflicted lacerations on her left forearm. The patient reported experiencing a lack of energy, difficulty falling asleep, and feeling overwhelmed following the birth of her third child one month earlier. She reported her depressive symptoms initially began with the birth of her second child four years ago but had dramatically worsened with the birth of her third child. The patient reported a sense of losing control of her life and feeling “mad about everything” in her life. Despite exhibiting symptomatology consistent with MDD with peripartum onset after the birth of her second child, the patient reported having never being diagnosed with it or receiving any treatment for her symptoms until the birth of her third child. When the patient’s symptoms worsened with the birth of her third child, she was started on bupropion XL 150 mg daily by her obstetrician but did not experience any improvement in her symptoms. No other interventions for the patient's symptoms were attempted. At the time of admission, the patient was positive for six of the DSM-5 criteria for MDD with peripartum onset, including depressed mood, insomnia, fatigue, agitation, reduced appetite, and suicidal ideation.

The patient’s social history appeared marginally contributory to her condition. She reported she was single and lived with a roommate and her three children. The patient’s support system consisted of her sister, mother, and newborn baby’s father, all of whom occasionally helped to take care of her newborn. The patient reported that her sister also had a history of MDD with peripartum onset but denied any other psychiatric family history. The patient had graduated from high school and was unemployed. The patient reported drinking socially and smoking four to five cigarettes per day. The patient also reported occasional cannabis use but denied the use of other recreational drugs. Prior to her admission, the patient had breastfed intermittently but had no plans to continue after discharge.

On her first day of hospitalization, the patient’s mental status examination was significant for a disheveled appearance and poor eye contact. The patient exhibited a depressed mood and a blunted affect. Her thought process was logical, and her thought content was negative for hallucinations, suicidal ideation, or homicidal ideation. After diagnosing the patient with MDD with peripartum onset, the treatment team discontinued the patient’s bupropion and started her on fluoxetine 20 mg daily. Additional treatment consisted of group therapy and daily team meetings.

During the second day of hospitalization, the patient appeared preoccupied with going home and was guarded throughout the interview. The patient also reported trouble falling asleep and required a dose of hydroxyzine for anxiety symptoms. The patient’s fluoxetine dose was increased to 40 mg once daily. During the third and fourth hospital days, the patient demonstrated increased response to treatment as evidenced by her improvement in mood and optimism about the future. Interestingly, the patient reported hearing someone call her name on the fourth day, but it was unclear whether this was a misinterpreted stimulus or a psychotic symptom.

On the fifth day of hospitalization, the patient reported marked improvement in her depressive symptoms and expressed an interest in returning home to her children. The patient reported she did not plan to continue breastfeeding. The patient endorsed a desire to continue treatment with a psychiatrist and therapist on an outpatient basis. On the sixth day of her hospitalization, having demonstrated the sustainability of her symptom improvement, the patient was discharged. At the time of discharge, the patient was stable, compliant with all medications, and had not exhibited any adverse effects from her medication. The patient was discharged with a prescription for fluoxetine 40 mg daily, a psychotherapy appointment, and an appointment with a psychiatrist.

## Discussion

This case demonstrates the need for further understanding of the current treatments for MDD with peripartum onset. Although treatment with an SSRI has been correlated with improved response rates and remission rates, the efficacy of treatment with both an SSRI and psychotherapy or psychotherapy alone is not well-characterized in the literature [7]. Furthermore, the relationship between the severity of the depression and the response to treatment with an antidepressant has not been established [7]. A deeper understanding of the indications for specific treatments could inform treatment guidelines and reduce initial treatment failure rates. This case also highlights the importance of understanding the role of chronicity when treating MDD with peripartum onset. Although traditionally symptoms are understood to present between pregnancy and four weeks postpartum, the nature and progression of symptomatology outside this period are poorly characterized. For instance, in this case, the patient had an insidious progression of her symptoms during the course of three pregnancies. In a similar vein, there is much to be learned about screening for MDD with peripartum onset. Screening with diagnostic assessment and engagement strategies has been shown to reduce the frequency of depressive episodes and improve recovery rates, but implementing these interventions in a manner that encourages compliance has been difficult [8,9].

For the patient described in this case report, comprehensive screening might have promoted earlier detection and treatment of her symptoms. Current screening recommendations from the American College of Obstetricians and Gynecologists (ACOG) consist of screening patients for depression once during the prenatal period and once during the postpartum visit [10]. More compendious screening for MDD with peripartum onset would likely result in health care savings as early intervention reduces the length of treatment and the need for more expensive treatments [9,11]. Additionally, using risk factors for MDD with peripartum onset, such as low levels of social support and a history of depression, could help with starting interventions early on with high-risk patients [12]. Finally, neurosteroids, such as brexanolone, are a promising approach to treating MDD with peripartum onset; further research into this class of compounds may lead to the discovery of more efficacious treatments for MDD with peripartum onset [13].

## Conclusions

MDD with peripartum onset is a disorder that has a significant effect on the lives of many mothers. The ACOG has created several guidelines for screening and treatment that attempt to mitigate the impact of the illness. Current screening measures and treatment recommendations are a move toward managing MDD with peripartum onset, but with mood symptoms continuing to impact many patients, there is still significant room for improvement.
